# Exploring a new mechanism between lactate and VSMC calcification: PARP1/POLG/UCP2 signaling pathway and imbalance of mitochondrial homeostasis

**DOI:** 10.1038/s41419-023-06113-3

**Published:** 2023-09-07

**Authors:** Yi Zhu, Jia-li Zhang, Xue-jiao Yan, Yuan Ji, Fang-fang Wang

**Affiliations:** 1 Department of Cardiology, The Affiliated Changzhou Second People’s Hospital of Nanjing Medical University, Changzhou Second People’s Hospital, Changzhou Medical Center, Nanjing Medical University, Changzhou, PR China; 2 Department of Gastroenterology Centre, The Affiliated Changzhou Second People’s Hospital of Nanjing Medical University, Changzhou, PR China

**Keywords:** Calcification, Mitophagy

## Abstract

Lactate leads to the imbalance of mitochondria homeostasis, which then promotes vascular calcification. PARP1 can upregulate osteogenic genes and accelerate vascular calcification. However, the relationship among lactate, PARP1, and mitochondrial homeostasis is unclear. The present study aimed to explore the new molecular mechanism of lactate to promote VSMC calcification by evaluating PARP1 as a breakthrough molecule. A coculture model of VECs and VSMCs was established, and the model revealed that the glycolysis ability and lactate production of VECs were significantly enhanced after incubation in DOM. Osteogenic marker expression, calcium deposition, and apoptosis in VSMCs were decreased after lactate dehydrogenase A knockdown in VECs. Mechanistically, exogenous lactate increased the overall level of PARP and PARylation in VSMCs. PARP1 knockdown inhibited Drp1-mediated mitochondrial fission and partially restored PINK1/Parkin-mediated mitophagy, thereby reducing mitochondrial oxidative stress. Moreover, lactate induced the translocation of PARP1 from the nucleus to the mitochondria, which then combined with POLG and inhibited POLG-mediated mitochondrial DNA synthesis. This process led to the downregulation of mitochondria-encoded genes, disturbance of mitochondrial respiration, and inhibition of oxidative phosphorylation. The knockdown of PARP1 could partially reverse the damage of mitochondrial gene expression and function caused by lactate. Furthermore, UCP2 was upregulated by the PARP1/POLG signal, and UCP2 knockdown inhibited Drp1-mediated mitochondrial fission and partially recovered PINK1/Parkin-mediated mitophagy. Finally, UCP2 knockdown in VSMCs alleviated DOM-caused VSMC calcification in the coculture model. The study results thus suggest that upregulated PARP1 is involved in the mechanism through which lactate accelerates VSMC calcification partly via POLG/UCP2-caused unbalanced mitochondrial homeostasis.

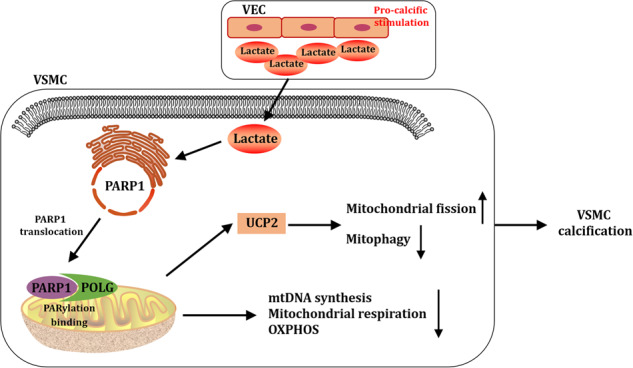

## Introduction

Vascular calcification is associated with an increased risk of heart disease, stroke, and atherosclerotic plaque rupture. Arterial calcification primarily occurs in the intima and media of arteries. Arterial intimal microcalcification (AIC) is associated with arterial stenosis and atherosclerotic plaque rupture, and arterial medial macrocalcification (AMC) can cause decreased vascular compliance and systolic hypertension and ultimately lead to diastolic dysfunction and heart failure [1]. Under the diabetic condition, arterial calcification is severe and extensive, leading to many problems for interventional therapy.

AMC is mainly driven by vascular smooth muscle cells (VSMCs), which express contractile phenotypic markers under normal physiological conditions. However, during inflammation, mitochondrial damage, oxidative stress, pro-apoptosis, and high glucose and phosphate conditions, VSMCs highly express bone morphogenetic protein 2 (BMP2) and runt-related transcription factor 2 (RUNX2) and then upregulate osteogenic proteins, resulting in osteogenic phenotype transformation [2]. Metabolomic assays suggest that the plasma lactate level is positively correlated with the Agatston score and the carotid intima–media thickness [3]. Moreover, a previous study showed that the plasma lactate levels of patients with diabetes were significantly higher than that of nondiabetic patients and that the plasma lactate levels of patients with severe coronary artery calcification were significantly higher than those of patients without calcification or with mild coronary artery calcification [4]. Furthermore, it was shown that exogenous lactate can damage the mitochondrial quality control system and promote the calcification of rat aorta and VSMCs [5, 6]. However, considering that lactate can freely cross the cell membrane, a large part of the lactate acting on VSMCs is derived from the adjacent vascular endothelial cells (VECs) [7]. Therefore, the construction of VSMC–VEC interaction model can better reflect the real cellular environment. In addition, whether lactate can promote VSMC calcification through other molecular mechanisms remains unclear.

Previous studies have shown that oxidative stress induced by mitochondrial dynamic changes and damage to the self-clearing mechanism plays an important role in arterial calcification [4, 8]. The integrity and normal replication of mitochondrial DNA (mtDNA) are crucial to the health of mitochondria [9]. MtDNA is synthesized by DNA polymerase γ (pol γ), which contains a 140-kDa catalytic subunit (encoded by POLG) and two 55-kDa process subunits (encoded by POLG2). Pol γ exhibits a DNA polymerase activity and 3′-5′ exonuclease activity and plays a physiological role with other mitochondrial proteins (such as 5′- 3′ DNA helicase, topoisomerase, RNA polymerase, and others) [10]. In a mouse model of vascular aging, the deletion of POLG destroyed the integrity of mtDNA and further accelerated vascular aging [11]. The vulnerability of mtDNA makes oxidative phosphorylation (OXPHOS) exposed to interference from external stimuli. Oxidative DNA damage is a relatively common endogenous damage. As an important effector protein, poly ADP-ribosyl transferases (PARPs) can simultaneously regulate the pathological characteristics of aging, inflammation, and death of calcified VSMCs [12]. The 113-kDa PARP1 protein (89-kDa active form) accounts for 85% of all PARP activity in cells. PARP1 plays the role of a transcriptional coactivator by direct binding or ADP-ribosylation enhancer and promoter, thereby regulating the expression of itself and other genes [13]. The overexpression of PARP1 can upregulate RUNX2, promoting the differentiation of VSMCs from contraction to the osteogenesis phenotype [14]. PARP1 knockout can inhibit the signal transducer and activator of transcription 1 (STAT1)/RUNX2 axis and reduce AIC in diabetes [15]. PARP1 is located in the nucleus; however, in the chronic infection model, it is highly expressed and translocated from the nucleus to the mitochondria and combines with POLG, resulting in the inhibition of POLG transcription and interference with the normal functioning of mtDNA and mitochondrial respiration [16].

Based on previous studies and existing literature, a VSMC–VEC coculture model was established to observe the effect of VEC lactate secretion on VSMC calcification in a high-glucose osteogenic culture environment. In addition, in VSMCs, the study explored the relationship among lactate, PARP1/POLG signal, mitochondrial fission/mitophagy, mtDNA integrity, and VSMC calcification as well as a new molecular mechanism between lactate and VSMC calcification.

## Materials and methods

### Ethical approval

All in vitro experiments were approved by the Animal Care and Research Committee of Nanjing Medical University, and all animal procedures were performed in accordance with the Guidelines of Animal Experiments from the Committee of Medical Ethics, the National Health Department of China (1998).

### Cell culture

Primary rat VSMCs were isolated from 6-week-old Sprague Dawley rat thoracic aortas (60–80 g, Experimental Animal Center in Nanjing, China) using the tissue explant method. Rat VECs were purchased from ScienCell (Shanghai, China). VSMCs/VECs were incubated in a 1:1 mixture of Dulbecco’s modified Eagle’s medium and Ham’s F12 medium with 10% fetal bovine serum (Gibco, USA) and antibiotics at 37 °C with 5% CO_2_. Cell calcification was induced with a diabetic osteogenic medium (DOM: comprising 33 mmol/L D-glucose, 50 μg/mL oxidized low-density lipoprotein, 2.5 mmol/L β-glycerophosphate, and 50 μg/mL ascorbic acid). The calcium deposition and osteoblastic phenotype markers were used for identification.

### VSMC–VEC coculture model

To investigate the effect of VEC secretion products on VSMC calcification, VECs were first treated with DOM or basic medium for 3 days (the best intervention condition in the previous experiment). During incubation, the cell culture medium was not changed. VSMCs were then added to the VEC culture and incubated for 7 days. This coculture was supplemented with serum and cell culture factors every other day.

### Calcium level measurement

Calcified VSMCs were solubilized in RIPA lysis buffer (Beyotime Biotechnology, Jiangsu, China), and the calcium level was measured using a calcium assay kit (Sigma-Aldrich, USA) and normalized to the total protein level measured with a bicinchoninic acid (BCA) protein assay kit (KeyGEN Biotechnology, Jiangsu, China).

### Alizarin red S staining

VSMCs were fixed in 4% paraformaldehyde for 30 min and then subjected to a series of treatments according to previous protocols(51). In alizarin red S staining, the calcium phosphate salts were visualized as a red stain.

### Transferase-mediated dUTP nick-end labeling (TUNEL) assay

The TUNEL method was performed using a detection kit (Roche, Germany) following the manufacturer’s instructions. VSMCs were fixed with 4% paraformaldehyde at room temperature, washed twice with PBS, and then permeabilized with 0.1% Triton X-100 for the FITC end-labeling of the fragmented DNA of apoptotic VSMCs. The TUNEL-positive cells were detected using confocal microscopy.

### Glucose uptake detection

Glucose uptake was detected using the fluorescent probe 2-[N-(7-nitrobenz-2-oxa-1,3-diazol-4-yl)amino]-2-deoxy-D-glucose (2-NBDG, Thermo Fisher Scientific, Shanghai, China). After washing, VSMCs were cultured with 100 μM 2-NBDG for 30 min in the dark, and fluorescence images were captured using a confocal microscope. At least six images per condition were analyzed.

### Lactate production measurement

The supernatant lactate levels were measured using commercial assay kits (Sigma-Aldrich) according to the manufacturer’s instructions and normalized to the total protein level determined using a BCA protein assay kit (KeyGEN Biotechnology).

### Extracellular acidification rate (ECAR) and oxygen consumption rate (OCR)

Seahorse XFe96 analyzer (Seahorse Bioscience, Boston, MA, United States) was used for measuring OCR and ECAR [16]. In brief, VSMCs were seeded into Seahorse 96-well plates and then treated according to the manufacturer’s instructions. For ECAR evaluation, glucose, oligomycin, and 2-deoxy-d-glucose (2-DG) were added according to the manufacturer’s instructions. Glycolysis = (last rate measurement before oligomycin injection)−(minimum rate measurement before glucose injection). Glycolytic capacity = (maximum rate measurement after oligomycin injection)−(minimum rate measurement after 2-DG injection). For OCR measurement, oligomycin, FCCP, and rotenone were added to the plates sequentially. Basal respiration = (last rate measurement before oligomycin injection)−(minimum rate measurement after rotenone injection). Maximal respiration = (maximum rate measurement after FCCP injection)−(minimum rate measurement after rotenone injection). All results were normalized to the cell number and determined at least thrice.

### Mitochondrial oxidative stress detection

Mitochondrial oxidative stress was determined via cardiolipin using nonyl-acrydine orange (NAO) staining (Genmed, Shanghai, China). Cardiolipin is sensitive to cell oxidation, i.e., the reduction or oxidation of the mitochondrial membrane mass decreases the fluorescence significantly. The red fluorescence intensity of mitochondria in VSMCs were used to determine the levels of mitochondrial oxidative stress.

### Activities of mitochondrial respiratory chain complexes

The activities of mitochondrial respiratory chain complex I–V (Genmed) were measured using a spectrophotometer (Thermo Fisher Scientific). The mitochondria of each group were separated, and 10 μg of mitochondrial protein was used for detection. The measurements were normalized to the total protein level. For complex I, total activity reading (TAR) = (Wave340 reading − Wave380 reading) 0 min − (Wave340 reading − Wave380 reading) 3 min; for complex II, TAR = Wave600 reading 0 min – Wave600 reading 1 min; for complex III, TAR = Wave550 reading 2 min – Wave550 reading 0 min; for complex IV, TAR = Wave550 reading 0 min – Wave550 reading 1 min; and for complex V, TAR = Wave340 reading 0 min – Wave340 reading 1 min.

### Cell transfection

To regulate LDHA, PARP1, POLG and uncoupling protein 2 (UCP2), lentiviral vectors, and puromycin-resistance selection genes were produced (Shanghai Genechem Co., Ltd.). VSMCs or VECs were first seeded into 6-well plates and cultured to 40–50% confluence. The cells were then transfected with the recombinant lentivirus (MOI = 5, 10, 20, 50, and 100). Stably transfected clones were selected after 2 weeks of puromycin selection treatment (0.5–1 μg/μL). The transfection efficiency was evaluated using western blotting.

To detect autophagic flux, VSMCs were transfected with GFP-mRFP-LC3 adenovirus (Shanghai Hanbio Biotechnology Co., Ltd.) for 4 h (MOI = 5). After transfection, the medium was replaced with complete medium for 12 h. At 48 h after transfection, autophagosomes (yellow dots) and autolysosomes (red dots) were detected under a confocal microscope (FV10i, Olympus, Japan).

### Mitochondria and lysosome morphology observation

VSMCs were cultured with a red mito-tracker and green lyso-tracker (Genmed Scientifics Inc.) for 20 min. Excess reagent was removed via PBS washes. Mitochondrial morphology was observed under a confocal microscope.

### Mitochondrial isolation

Mitochondrial fractions were obtained using a mitochondrial isolation kit (Thermo Fisher Scientific).

In brief, after VSMCs were incubated in ice-cold mitochondrial lysis buffer for 15 min, the cell suspension was homogenized through 20 strokes. Next, the homogenate was centrifuged at 1000 ×*g* for 10 min at 4 °C to remove the nuclei and unbroken cells. The supernatant was then collected and centrifuged again at 11000 ×*g* for 10 min at 4 °C to obtain the mitochondrial fraction. For western blotting, the mitochondrial fractions were stored in mitochondrial lysis buffer containing PMSF.

### Western blot analysis

VSMCs were lysed in RIPA lysis buffer, and the protein concentration was measured using the BCA protein assay kit. Subsequently, proteins (20-40 μg) were separated by SDS-PAGE and then transferred to PVDF membranes. The membranes were blocked with 5% bovine serum albumin for 1.5 h and then incubated overnight at 4 °C with the following primary antibodies: Anti-transgelin (TAGLN) (CST52011) (1:1000), anti-Cleaved-Caspase3 (CST9661) (1:1000), anti-Bcl-2 (CST3498) (1:1000), and anti-Bax (CST14796) (1:1000) antibodies were obtained from Cell Signalling Technology (Danvers, MA, USA). Anti-RUNX2 (ab76956) (1:1000), anti-BMP-2 (ab214821) (1:1000), anti-dynamin related protein 1 (Drp1) (ab184247) (1:1000), anti-PTEN induced kinase 1 (PINK1) (ab186303) (1:2000), anti-Parkin (ab77924) (1:2000), anti-voltage dependent anion channel 1 (VDAC1) (ab15895) (1:3000), anti-PARP1 (ab191217) (1:1500), anti-LaminB1 (ab16048) (1:3000), anti-POLG (ab128899) (1:1000), anti-UCP2 (ab67241) (1:1000) antibodies were purchased from Abcam (Cambridge, MA, USA). Anti-PAR antibody (4335-MC-100) (1:1000) was purchased from Trevigen (MD, USA). The membranes were then incubated for 1 h at room temperature with horseradish peroxidase-conjugated secondary antibodies (anti-rabbit or anti-mouse IgG) (Sigma). The proteins were visualized using electrochemiluminescence, and the results were quantified with Image-Pro Plus 6.0 software.

### Co-immunoprecipitation (co-IP)

The co-IP method was performed as previously described [17]. In brief, human umbilical vein endothelial cells (HUVECs) were lysed using western blot and IP buffer (Thermo Fisher Scientific). The whole-cell extract was used as input, and the remainder was incubated with the PARP1 antibody (1:50) or POLG antibody (1:50) at 4 °C overnight with slow rotation. Then, 40 μL Protein A/G magnetic beads (Thermo Fisher Scientific) were added and the lysates were incubated for 3 h. After PBS washing, the proteins were eluted by boiling the beads. The eluted proteins and input extracts were analyzed using western blotting.

### RT-qPCR

RNA isolation and RT-qPCR were performed according to previously described protocols [6]. Briefly, after total RNA extraction from HUVECs using TRIzol reagent (Sigma), cDNA was generated by RT-PCR. RT-qPCR was carried out on a StepOnePlus Real-Time PCR System (Thermo Fisher Scientific) using the primers listed in Table 1. All mRNA levels were normalized to β-actin or 18 S RNA expression levels and determined using the ΔΔCt method.

**Table 1:** Primer sequences for the RT-qPCR analysis.

Genes	Primer sequences
RUNX2	Forward 5′-GAGCGGACGAGGCAAGAGTTTC-3′
	Reverse 5′-GCGTGGTGGAATGGATGGATGG-3′
BMP2	Forward 5’-AAGCGTCAAGCCAAACACAAACAG-3'
	Reverse 5’-CAACCCTCCACAACCATGTCCTG-3'
TAGLN	Forward 5’-TTCCAGCCCACAAACGACCAAG-3'
	Reverse 5’-ATCACGCCATTCTTCAGCCACAC-3'
ND4	Forward 5’-ACGCACTCACAGTCGCATCATAATC-3'
	Reverse 5’-GGGTTGAGGGATAGGAGGAGAATGG-3'
COI	Forward 5’-CCTGTGTTGGCTGGGGCTATTAC-3'
	Reverse 5’-GCTCACACCACACAACCAATCAAAC-3'
CYTB	Forward 5’-CTCAGTAGACAGTCCCACCCTCAC-3'
	Reverse 5’-GACGGATCGGAGAATTGTGTAGGC-3'
ATP6	Forward 5’-ACAACTAACCTCCTCGGACTCCTG-3'
	Reverse 5’-CGTAGGCTTGGATTAAGGCGACAG-3'
ATP8	Forward 5’-ACACCAAAAGGACGAACCTG-3'
	Reverse 5’-AGAATTACGGCTCCTGCTCA-3'
TOP1	Forward 5’-AGAGAGCGGATTGCCAACTTCAAG-3'
	Reverse 5’-TCCAGGAGACCAGCCAAGTAACC-3'
POLRMT	Forward 5’-CTGAACTGCTGGAAGTGCTGGAG -3'
	Reverse 5’-GTCTCTACCGCCTTGGCTTTCG-3'
SSBP1	Forward 5’-GGAGTCTGAAGTAGCCAGCAGTTTG-3'
	Reverse 5’-TTCACCATAGTCCACTTTGCCTTCC-3'
POLG1	Forward 5’-CACTACGGACGAACTCATACCACTG-3'
	Reverse 5’-GATGCACCTCCACCAGACTGTTAAG-3'
TFB1M	Forward 5’-CGCGTGTGTATGTAGGTCTCATGG-3'
	Reverse 5’-CCACCAGAAGCTCGGCAATATCAG-3'

### Statistical analysis

All data are expressed as the mean ± standard deviation for approximately normally distributed data and as the medians (interquartile range) for skewed continuous variables. Comparisons of continuous variables in two groups were performed using Student’s t-tests. One-way ANOVA with post-hoc comparisons was used for multiple group comparisons. Receiver operating characteristic (ROC) analysis was performed to determine whether the explored variables were predictive of CAD. Two-sided *P*-values < 0.05 were considered to indicate statistical significance. Statistical analyses were performed using SPSS software 22.0. All experiments were repeated independently at least three times.

## Results

### DOM culture enhanced glycolysis ability and lactate production in VECs

**Fig. 1:**
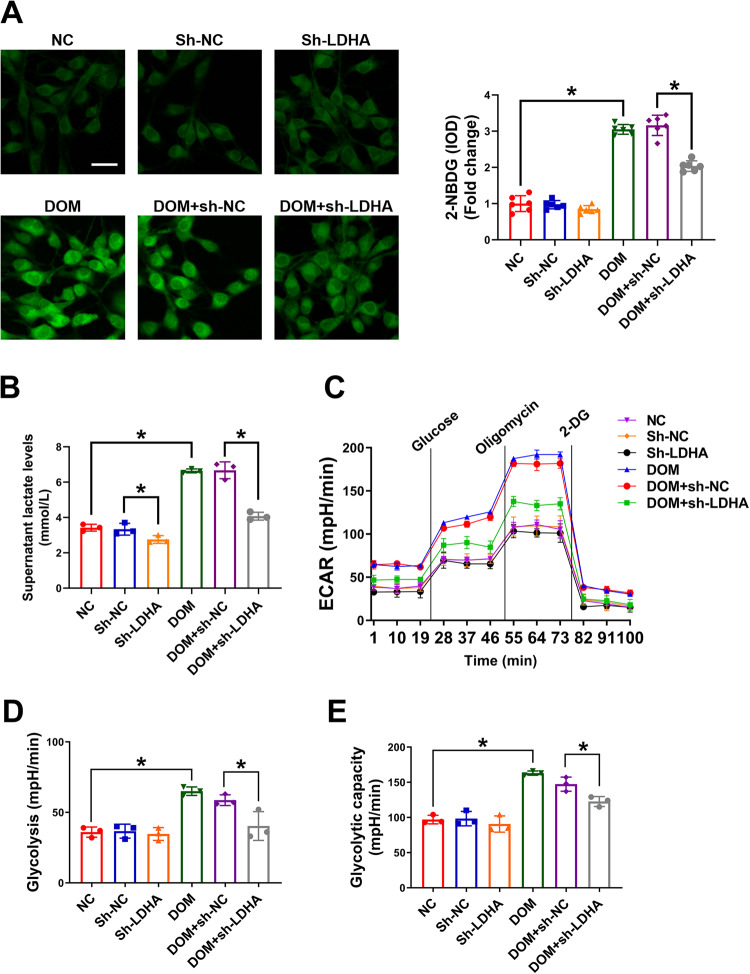
DOM culture enhanced glycolysis ability and lactate production in VEC. VECs (transfected with or without sh-LDHA) were cultured with DOM or basic medium for 3 days. **A** The glucose uptake was detected with 2-NBDG assay. At least 6 images per condition were analyzed. Scale bar, 10 μm. **B** Supernatant lactate levels were measured with the lactate assay kits. At least 3 repeats per condition were analyzed. **C**–**E** Glycolysis and glycolysis capacity were determined by ECAR. At least 3 repeats per condition were analyzed. *p < 0.05 versus the indicated treatment.

Since only approximately 2–6% of the organelles are mitochondria in VECs), the energy generation of VECs mainly depends on glycolysis, thereby a large amount of lactate are generated [18]. Changes in the glucose metabolism of VECs in the high glucose osteogenic culture environment were first observed. As shown in Fig. 1A, the glucose uptake of cells increased significantly after DOM intervention, suggesting that glucose metabolism is enhanced and glucose uptake is reduced significantly after LDHA knockdown. Then, the lactate production and glycolysis ability of VECs were analyzed. As shown in Fig. 1B–E, the lactate production and glycolysis capacity of cells significantly increased after DOM intervention and LDHA knockdown significantly inhibited the lactate production and glycolysis capacity. The above results suggest that under the high glucose and osteogenesis environment, the lactate production of VECs is increased and LDHA knockdown can block the glycolytic flux toward lactate to a certain extent.

### Lactate secreted by VECs promoted VSMC calcification

**Fig. 2:**
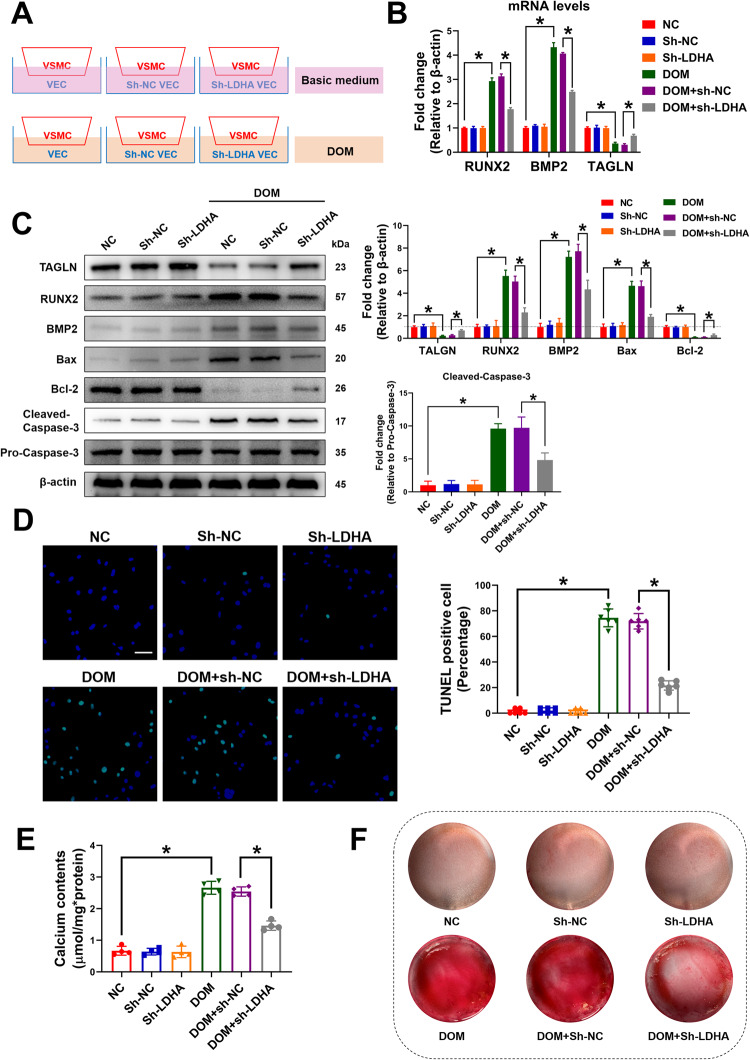
Lactate secreted by VEC promoted VSMC calcification. VECs (transfected with or without sh-LDHA) were cultured with DOM or basic medium for 3 days, then VSMCs were added to the co-culture model on the basis of VEC culture for 7 days. **A** The VSMC-VEC co-culture model. **B** The mRNA levels of RUNX2, BMP2 and TAGLN were detected by RT-qPCR. At least 3 repeats per condition were analyzed. **C** The protein levels of BMP2, RUNX2, TAGLN, Bax, Bcl-2 and cleaved-caspase3 were detected by western blotting. Representative bands are displayed (*n* = 3 independent experiments). **D** The TUNEL positive cells were observed using confocal microscopy. At least 6 images per condition were analyzed. Scale bar, 50 μm. **E** The calcium content in VSMCs were determined using calcium content assay kits. At least 3 repeats per condition were analyzed. **F** The calcium nodules were stained with Alizarin red S. *p < 0.05 versus the indicated treatment.

To investigate the effects of lactate secreted by VECs on VSMC calcification, a VSMC–VEC coculture model was established (Fig. 2A). First, the expression of contractile and osteogenic phenotypic molecules (TAGLN, RUNX2, and BMP2) was detected in VSMCs. As shown in Fig. 2B, C, VEC medium (DOM intervention) could significantly upregulate RUNX2 and BMP2 in VSMCs and down-regulate TAGLN. After knocking-down LDHA in VECs, the expression of RUNX and BMP2 in VSMCs decreased and that of TAGLN increased. Then, the calcium deposition level in VSMCs was detected and alizarin red S staining in VSMCs was performed. As expected, the calcium salt deposition (Fig. 2E) and calcium nodule formation (Fig. 2F) in VSMCs were increased after DOM exposure and LDHA knockdown inhibited calcium deposition in VSMCs. As the osteogenic phenotype transformation of VSMCs is usually accompanied by apoptosis under the intervention of high glucose and phosphate, apoptosis in VSMCs was then evaluated. As shown in Fig. 2C, D, DOM intervention increased the expression of apoptosis-related proteins and TUNEL-positive cells. After knocking-down LDHA, VSMC apoptosis was inhibited. These results suggest that lactate secreted by VECs can promote VSMC osteogenesis phenotype transformation and induce VSMC apoptosis.

### PARP1 was involved in lactate-induced overactivation of mitochondrial fission and inhibition of mitophagy

**Fig. 3:**
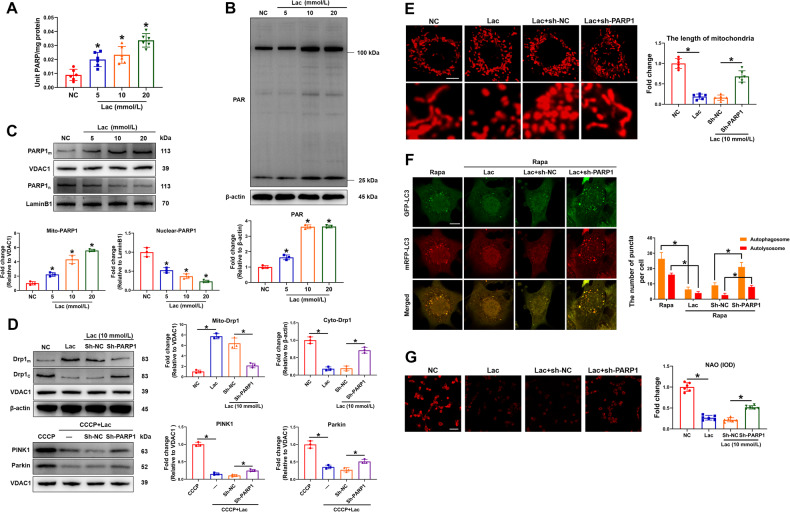
PARP1 was involved in lactate-induced overactivation of Drp1-related mitochondrial fission and inhibition of PINK1/Parkin-mediated mitophagy. **A** PARP activity in VSMCs was determined using the universal colorimetric PARP assay kit. At least 6 repeats per condition were analyzed. **B** Representative western blot analysis of PARylation in VSMCs. Representative bands are displayed (*n* = 3 independent experiments). **C** The protein levels of mito-PARP1 and nuclear-PARP1 were detected by western blotting. Representative bands are displayed (*n* = 3 independent experiments). **D** The protein levels of mito-Drp1, cyto-Drp1, PINK1 and Parkin were detected by western blotting. Representative bands are displayed (*n* = 3 independent experiments). **E** VSMCs were stained with mito-tracker red to evaluate the mitochondrial fragmentation. The length of mitochondria was measured. At least 6 cells per condition were imaged. Scale bar, 2 μm. **F** VSMCs were pre-treated with Rapa (10 μM) for 4 h. Then VSMCs were transfected with mRFP-GFP-LC3 adenovirus for 4 h for fluorescent analysis. The yelllow puncta indicate autophagosome, and the free red puncta indicate autolysosome. Scale bar, 10 μm. At least 6 cells per condition were imaged. Scale bar, 2 μm. **G** Mitochondrial oxidative stress was detected by NAO probe. At least 6 images per condition were analyzed. Scale bar, 50 μm. *p < 0.05 versus the indicated treatment.

Oxidative stress and apoptosis are induced in vascular calcification, and PARP1 can be activated by multiple cellular stresses, particularly stress state. Thus, it was speculated that PARP1 could be evoked during vascular calcification. Compared with normal controls, the total poly(ADP-ribosyl)ation levels and PARP activity were significantly increased after lactate intervention (Fig. 3A, B). The level of PARP1 in the nucleus and mitochondria, respectively, was then detected, and as shown in Fig. 3C, with the increase in the concentration of lactate intervention, the expression of PARP1 in the nucleus decreased while that in the mitochondria increased, suggesting the translocation of PARP1 from the nucleus to the mitochondria. A previous study showed that lactate can induce the translocation of Drp1 from the cytoplasm to mitochondria, thereby activating the Drp1-mediated mitochondrial fission, inhibiting mitophagy, and finally inducing mitochondria-related apoptosis [5]. As PARP1 translocated from the nucleus to the mitochondria, it was speculated that PARP1 plays a role as a downstream regulatory factor of lactate.

As shown in Fig. 3D, E, lactate induced the translocation of Drp1 from the cytoplasm to mitochondria, resulting in short rod-like changes in several mitochondria in VSMCs, suggesting the occurrence of mitochondrion fission. PARP1 knockdown inhibited the translocation of Drp1 and subsequent mitochondrial fission to a certain extent. Then, the relationship between PARP1 and mitophagy was tested; CCCP was used to induce mitophagy. Previous research has suggested that PARP1 is closely related to the PINK1/Parkin-mediated mitophagy; [19] thus, only the relationship between PARP1 and PINK1/Parkin-mediated mitophagy was evaluated. The results showed that lactate downregulated the increase in PINK1/Parkin expression induced by CCCP. After the knockdown of PARP1, the level of PINK1/Parkin was partially restored, suggesting that PARP1 is involved in the regulation of PINK1/Parkin-mediated mitophagy by lactate (Fig. 3D). The GFP-mRFP-LC3 double-labeled adenovirus was then used to observe the overall intracellular autophagy. As shown in Fig. 3F, after rapamycin intervention, a large number of autophagosomes (yellow dots) and autolysosomes (red dots) were generated in VSMCs and the production of autophagosomes and autolysosomes was inhibited by lactate. The knockdown of PARP1, however, partially restored the production of autophagic autophagosomes and autolysosomes based on the intervention of lactate, which further suggests that lactate regulates mitophagy via PARP1. Finally, the relationship between PARP1 and mitochondrial oxidative stress was observed. As shown in Fig. 3G, NAO probe was used to detect the mitochondrial oxidative stress level in VSMCs. The fluorescence intensity of NAO was inversely proportional to the mitochondrial oxidative stress level. Lactate significantly reduced the fluorescence intensity of NAO in VSMCs and PARP1 knockdown reversed this process, which suggests that lactate induces mitochondrial oxidative stress via PARP1. Thus, it can be concluded that PARP1 is involved in the lactate-induced overactivation of Drp1-induced mitochondrial fission and inhibition of PINK1/Parkin-mediated mitophagy.

### Knockdown of PARP1 preserved mtDNA level and OXPHOS

**Fig. 4:**
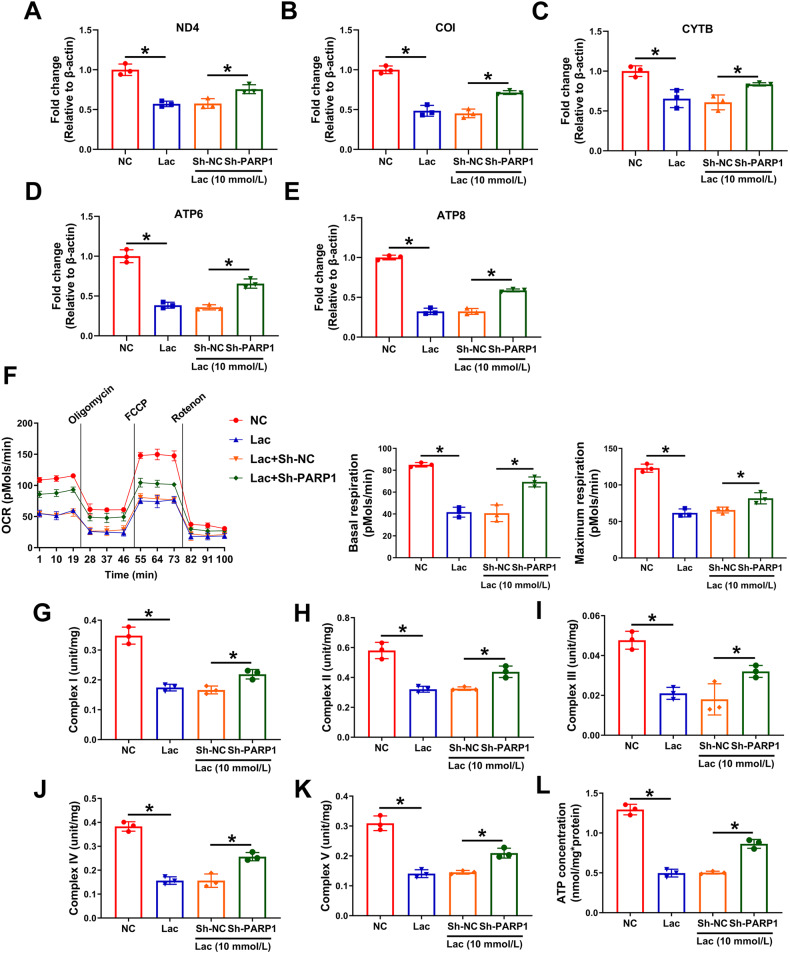
Knockdown of PARP1 preserved mtDNA content and OXPHOS. **A**–**E** The mRNA levels of ND4, COI, CYTB, ATP6 and ATP8 were detected by RT-qPCR. At least 3 repeats per condition were analyzed. **F** The OCR assay was used to observe the mitochondrial respiratory function, and basal and maximum respiration were determined. At least 3 repeats per condition were analyzed. **G**–**L** Electron transport chain operation in VSMCs was detected by mitochondrial respiratory chain complex I–V activities. At least 3 repeats per condition were analyzed. *p < 0.05 versus the indicated treatment.

As PARP1 is a DNA repair enzyme and mtDNA encodes the essential components of the mitochondrial respiratory chain [20], whether the mitochondrial translocation of PARP1 preserved mtDNA in VSMC calcification was evaluated. As shown in Fig. 4A–E, the protection of mtDNA level after PARP1 knockdown was associated with the preservation of mtDNA-encoded gene expression at mRNA (ND4, COI, CYTB, ATP6, and ATP8) levels, suggesting that mtDNA level is decreased by lactate via PARP1. Then, the relationship between PARP1 and mitochondrial respiration and OXPHOS was analyzed. By detecting OCR and mitochondrial respiratory chain complex I–V activities, lactate was observed to disturb mitochondrial respiration and reduce mitochondrial respiratory chain complex I–V activities; PARP1 knockdown could partially restore mitochondrial respiration and mitochondrial respiratory chain complex I–V activities (Fig. 4F–L). These results indicate that PARP1 knockdown can preserve mtDNA level and OXPHOS.

### PARP1 bound to POLG in lactate-caused VSMC calcification

**Fig. 5:**
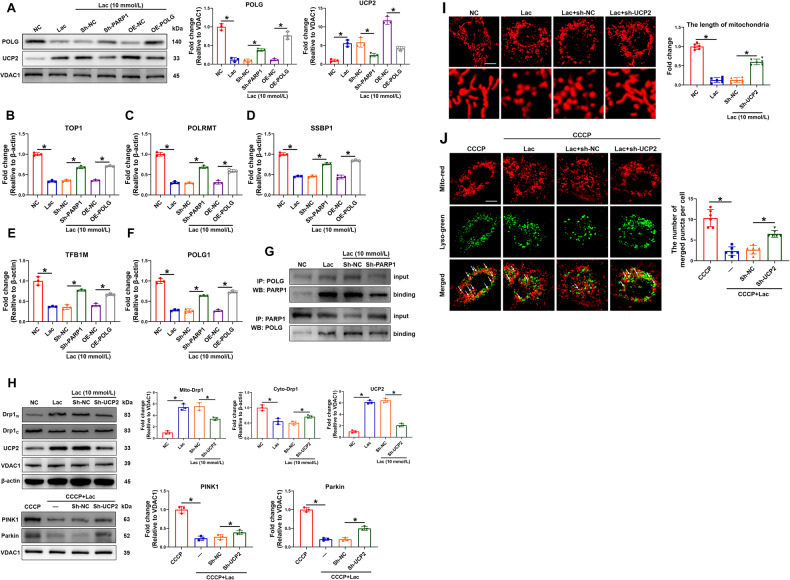
UCP2-regulated mitochondrial fission and mitophagy was affected by PARP1/POLG signal. **A** The protein levels of POLG and UCP2 were detected by western blotting. Representative bands are displayed (*n* = 3 independent experiments). **B**–**F** The mRNA levels of TOP1, POLRMT, SSBP1, TFB1M, and POLG1 were detected by RT-qPCR. At least 3 repeats per condition were analyzed. **G** Co-IP assay was performed to confirm the binding between PARP1 and POLG. **H** The protein levels of mito-Drp1, cyto-Drp1, UCP2, PINK1 and Parkin were detected by western blotting. Representative bands are displayed (*n* = 3 independent experiments). **I** VSMCs were stained with mito-tracker red to evaluate the mitochondrial fragmentation. The length of mitochondria was measured. At least 6 cells per condition were imaged. Scale bar, 2 μm. **J** VSMCs were stained with mito-tracker red and lyso-tracker green to observe the merge between mitochondria and lysosome. The number of merged puncta was measured. At least 6 cells per condition were imaged. Scale bar, 2 μm. *p < 0.05 versus the indicated treatment.

As mtDNA level was improved after PARP1 knockdown, it was considered that PARP1 constitutes a risk factor by adversely affecting mtDNA replication. The mRNA levels of the components of the mtDNA replication/repair complex, including those of TOP1, POLRMT, and SSBP1, as well as the genes of the mitochondrial transcription machinery, including TFB1M, were significantly decreased after lactate incubation. However, PARP1 knockdown elevated the mRNA levels of TOP1, POLRMT, SSBP1, and TFB1M (Fig. 5B–E), suggesting that PARP1 knockdown preserved mtDNA integrity. As POLG is essential to the integrity of mtDNA, the effects of lactate and PARP1 knockdown on POLG expression were next investigated. As expected, lactate intervention markedly decreased the mRNA and protein levels of POLG, which could be reversed by PARP1 knockdown (Fig. 5A, F). Next, POLG was overexpressed via cell transfection; the transfection efficiency was almost 80%. The results showed that the overexpression of POLG increased the mRNA levels of TOP1, POLRMT, SSBP1, and TFB1M (Fig. 5B–E). To further explore the interaction between PARP1 and POLG, a co-IP assay of PARP1 and POLG was performed, and the results confirmed that PARP1 and POLG have a combination (Fig. 5G). These results suggest that PARP1 binds to POLG and suppresses the POLG-mediated mtDNA synthesis during lactate-caused VSMC calcification.

### UCP2-regulated mitochondrial fission and mitophagy was affected by PARP1/POLG signal

As UCP2 is closely related to mitochondrial function decompensation, whether UCP2 is involved in the PARP1-mediated imbalance of mitochondrial homeostasis was explored [21]. As shown in Fig. 5A, the protein level of UCP2 was significantly increased in the lactate intervention group compared with that of the normal control group, whereas PARP1 knockdown reduced UCP2 expression. POLG overexpression elevated the UCP2 protein level under lactate. The relationship between UCP2 and Drp1-mediated mitochondrial fission and PINK1/Parkin-mediated mitophagy was then explored. Under the intervention of lactate, the knockdown of UCP2 inhibited the translocation of Drp1 from the cytoplasm to mitochondria (Fig. 5H). The observation of mitochondrial morphology under confocal microscope also confirmed that the knockdown of UCP2 delayed mitochondrial fission caused by lactate (Fig. 5I). In addition, the knockdown of UCP2 upregulated the expression of PINK1 and Parkin (Fig. 5H). The colocalization of mitochondria and lysosomes showed that lactate can inhibit the fusion of mitochondria and lysosomes, whereas the knockdown of UCP2 can induce the fusion of mitochondria and lysosomes (Fig. 5J), suggesting that UCP2 regulates PINK1/Parkin-mediated mitophagy.

### Knockdown of UCP2 in VSMCs alleviated VSMC calcification caused by VEC-secreted lactate

**Fig. 6:**
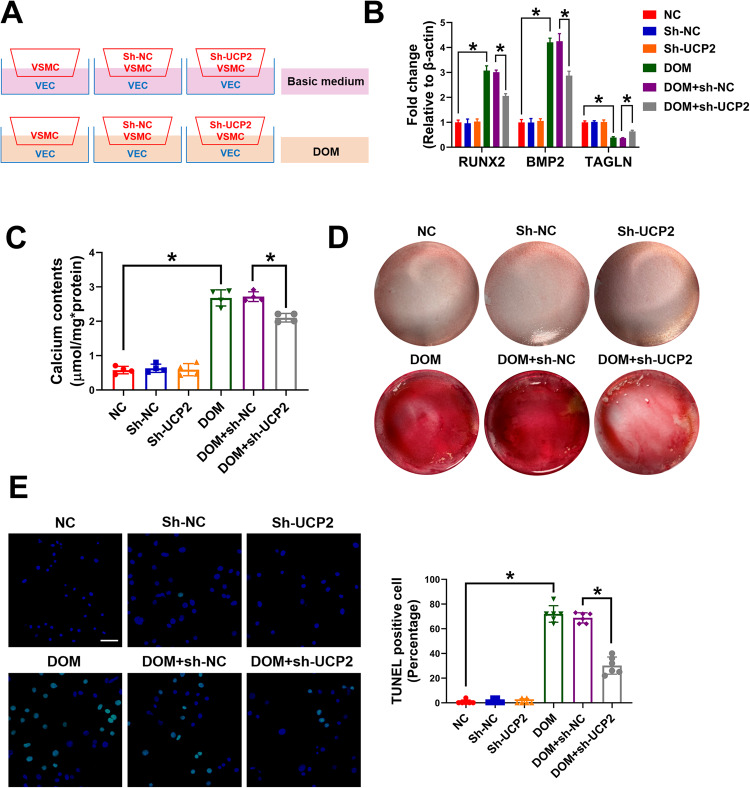
Knockdown of UCP2 in VSMC alleviated VSMC calcification caused by VEC secreted lactate. VECs were cultured with DOM or basic medium for 3 days, then VSMCs (transfected with sh-NC or sh-UCP2) were added to the co-culture model on the basis of VEC culture for 7 days. **A** The VSMC-VEC co-culture model. **B** The mRNA levels of RUNX2, BMP2, and TAGLN were detected by RT-qPCR. At least 3 repeats per condition were analyzed. **C** The calcium content in VSMCs was determined using calcium content assay kits. At least 3 repeats per condition were analyzed. **D** The calcium nodules were stained with Alizarin red S. **E** The TUNEL positive cells were observed using confocal microscopy. At least 6 images per condition were analyzed. Scale bar, 50 μm. *p < 0.05 versus the indicated treatment.

Finally, the role of UCP2 in VSMC calcification was explored. A VSMC–VEC coculture model was established and UCP2 was knocked-down in VSMCs (Fig. 6A). Compared with the control groups, the levels of osteogenic protein, calcium deposition, and degree of calcium nodule staining in UCP2 knockdown groups were significantly reduced (Fig. 6B–D). TUNEL staining confirmed UCP2 knockdown could reduce VSMC apoptosis induced by lactate (Fig. 6E). Therefore, these results suggest that UCP2 is an important molecule that participates in lactate-caused VSMC calcification.

## Discussion

In the present study, the following results were obtained. Lactate secreted by VECs promoted VSMC calcification, and it caused the overactivation of Drp1-related mitochondrial fission and suppression of PINK/Parkin-mediated mitophagy through the PARP1/POLG/UCP2 axis. In addition, lactate induced the translocation of PARP1 from the nucleus to mitochondria, which bound with POLG to inhibit mtDNA synthesis, mitochondrial respiration, and OXPHOS. Finally, UCP2 knockdown alleviated lactate-induced VSMC calcification.

Arterial calcification is the common pathological manifestation of atherosclerosis, diabetes angiopathy, end-stage renal disease, hypertension, and other diseases as well as aging. Arterial calcification is an independent risk factor for the increased risk of clinical cardiovascular and cerebrovascular adverse events [1]. Arterial calcification in diabetes is severe and widespread, causing many problems for interventional therapy. At present, there is no effective method to delay the occurrence of arterial calcification in diabetes. Therefore, it is important to explore the pathogenesis and intervention strategies of arterial calcification in diabetes.

Glucose is the main source of energy for VSMCs. Approximately 30% of ATP in VSMCs is produced by glycolysis, and about 90% of glycolysis flux is converted into lactate. Lactate can freely penetrate the cell membrane via the monocarboxylate transporter ¼ [18, 22]. The community atherosclerosis risk factors study showed that the plasma lactate level of patients with diabetes was higher than that of people without diabetes. At the same time, in those without diabetes, higher lactate level was associated with higher fasting blood glucose [23]. The present study further evaluated the relationship between lactate and arterial calcification. The plasma lactate level and the expression of key glycolytic enzymes in the arterial median tissue of rats with arterial calcification were significantly higher than those of rats without arterial calcification. In addition, lactate intervention (ex vivo) aggravated aortic calcification in rats. At the cellular level, the intracellular and secreted lactate levels of calcified VSMCs were significantly higher than those of noncalcified VSMCs. After inhibiting the production of lactate, the calcification of VSMCs was alleviated. Exogenous lactate destroyed the mitochondrial quality control system through p53 and promoted the osteogenic phenotype transformation of VSMCs [4–6]. In addition, the calcification of HUVECs was accompanied by abnormal glucose metabolism, i.e., the accumulation of lactate [24]. Considering that VECs and VSMCs are an entire ecological site, VEC damage must act on VSMCs through a paracrine manner. Thus, a coculture model of VECs and VSMCs was established, which showed that VECs secrete a large amount of lactate under high-glucose and high-phosphate environment and that lactate could directly cause VSMC calcification. Therefore, the lactate acting on VSMCs is derived from its own production on the one hand and the paracrine action of adjacent cells on the other. In atherosclerosis and arteriosclerosis models, the influence of macrophages on VSMCs cannot be ignored. Previous research also confirmed that macrophages promote VSMC calcification through galectin-3 [25]. In the future, the relationship among macrophages, lactate, and VSMCs will be explored.

Studies have confirmed that PARP1 is an important action protein and effector protein of vascular calcification. It plays a role of transcriptional coactivator and regulates the expression of itself and other genes through the direct binding of ADP-ribosylation enhancer and promoter [13]. The overexpression of PARP1 can upregulate RUNX2, accelerate the differentiation of VSMCs from contraction to the osteogenesis phenotype, and promote the expression of mineralization regulatory protein and calcium deposition [14]. PARP1 knockout can inhibit the STAT1/RUNX2 axis and reduce AIC in diabetes [15].

PARP1 is located in the nucleus; however, in the chronic infection model, PARP1 is highly expressed and translocated from the nucleus to mitochondria, binds with POLG, inhibits POLG transcription, and interferes with the normal function of mtDNA and mitochondrial respiratory function [16]. The vulnerability of mtDNA makes it sensitive to external stimuli, resulting in damage to the activity of electron transfer chain and reduction of ATP production. The present study results showed that lactate directly interfered with mtDNA synthesis and then affected mitochondrial respiration and OXPHOS. In addition, PARP1 reduced the mtDNA damage effect caused by lactate, suggesting that PARP1 is an important molecular target for lactate to damage mtDNA. In addition, PARP1 relied on POLG to regulate the replication of mtDNA, which is also consistent with previous reports. The shortage of cell energy directly leads to the damage of cell organelles and phenotypic transdifferentiation, leading to programmed cell death [26]. Whether it is cell apoptosis [27], necrosis [28], pyroptosis [29], autophagic death [30], or ferroptosis [31], which is accompanied by mitochondrial energy metabolism damage, the dead cell body will be an important binding site for calcium salt deposition [32]. Therefore, from a macro perspective, the accumulation of lactate indicates the ineffective output of the energy source glucose, and the subsequent effect of lactate further aggravates the energy shortage in cells. Of course, researchers have suggested that lactate is more likely to be a general fuel than glucose [33]. However, the physiological state and pathological state are quite different. The complexity of pathological injury factors implies that the compensation and decompensation of energy products are in a state of transition at any time. As a pathological effector protein, PARP1 is similar to a direct regulator of cell energy shortage.

In addition to the damage effect of PARP1/POLG on mtDNA, the PARP1/POLG signal was found to upregulate UCP2, thereby overactivating Drp1-mediated mitochondrial fission and inhibiting PINK1/Parkin-mediated mitophagy. As an important part of the mitochondrial quality control system, the normal operation and coordination of mitochondrial fission and mitophagy can protect the integrity of the inner and outer membrane structure of mitochondria, thereby maintaining the integrity of mtDNA and mitochondrial membrane potential [34, 35]. Once the mitochondrial structure is damaged and the damaged mitochondria cannot clear themselves in time, the damaged mitochondria will produce an ROS storm, destroy the redox balance, and then lead to cell death [36]. However, it is still unclear as to how PARP1/POLG regulates UCP2. Mitochondrial UCPs are members of the larger family of mitochondrial anion carrier proteins. UCPs separate oxidative phosphorylation from ATP synthesis with energy dissipated as heat, which is referred to as the mitochondrial proton leak. UCPs facilitate the transfer of anions from the inner to the outer mitochondrial membrane and the return transfer of protons from the outer to the inner mitochondrial membrane. Moreover, they reduce the mitochondrial membrane potential in mammalian cells [37, 38]. Thus, UCP2 plays an important role in maintaining the stability of the mitochondrial membrane potential and ATP production. Therefore, in the present study, changes in UCP2 and POLG were at the same level. The regulation of PARP1 on UCP2 likely depends on mtDNA damage, resulting in the compensatory upregulation of UCP2, and the subsequent changes in mitochondrial fission and mitophagy depend on the compensatory upregulation of UCP2.

It can be thus concluded that under the intervention of lactate, PARP1 translocates from the nucleus to mitochondria, binds with POLG, and inhibits POLG-mediated mtDNA synthesis and OXPHOS, thereby causing the compensatory upregulation of UCP2. These processes damage the mitochondrial quality control, further damaging the mitochondria and ultimately leading to mitochondrial oxidative stress and cell apoptosis.

The present study has some limitations. First, the interaction between VSMCs and VECs could not be verified at the animal level. Indeed, the specific knockout of functional molecules in VSMCs or VECs will further support the conclusion of this study. Second, no evidence of the relationship between POLG and UCP2 was found. Proteomics and metabolomics may provide some clues regarding this relationship.

## Conclusions

In this study, lactate was found to promote VSMC calcification through PARP1. PARP1 then translocated from the nucleus to mitochondria to bind with POLG, thereby inhibiting mtDNA synthesis, mitochondrial respiration, and OXPHOS. Moreover, lactate upregulated UCP2 through PARP1/POLG signal, overactivating Drp1-related mitochondrial fission and suppressing PINK/Parkin-mediated mitophagy. Finally, UCP2 knockdown alleviated lactate-induced VSMC calcification.

## Supplementary information

Reproducibility checklist

Full and uncropped western blots

## Data Availability

The data that support the findings of this study are available from the corresponding authors upon reasonable request.
