# Supplementary material for: Exploring a new mechanism between lactate and VSMC calcification: PARP1/POLG/UCP2 signaling pathway and imbalance of mitochondrial homeostasis
Source: Cell Death Dis. 2023 Sep 7;14(9):598. doi: 10.1038/s41419-023-06113-3 (PMC10484939; doi:10.1038/s41419-023-06113-3)

Figure 2

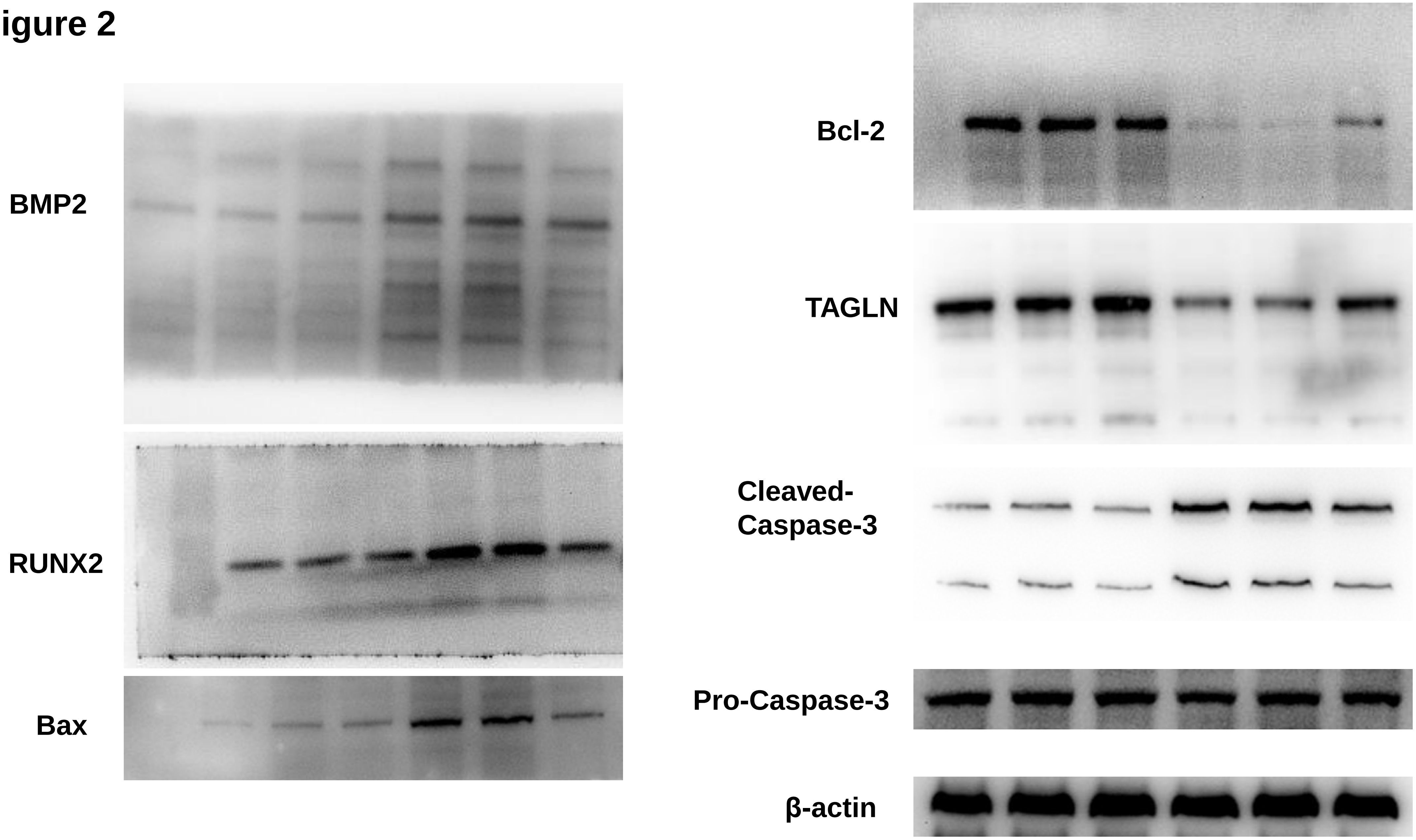
BMP2
RUNX2
Bax
Bcl-2
TAGLN
Cleaved-
Caspase-3
Pro-Caspase-3
β-actin

Figure 3B

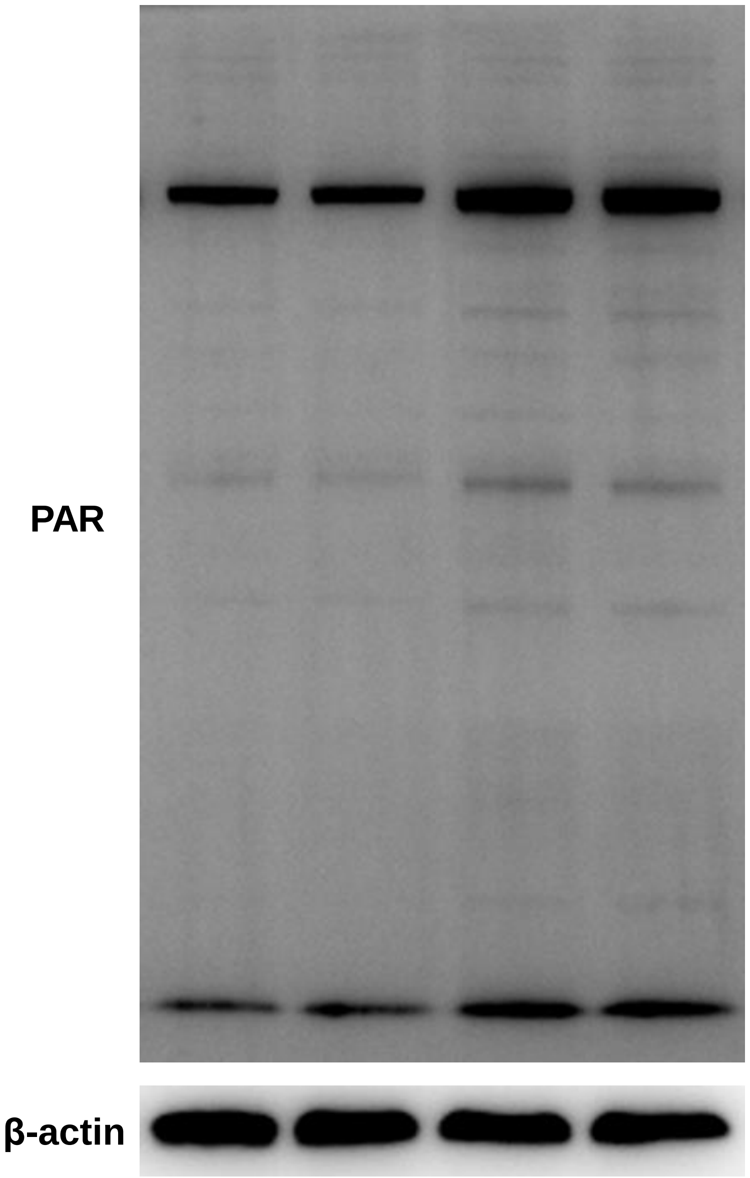

Figure 3C

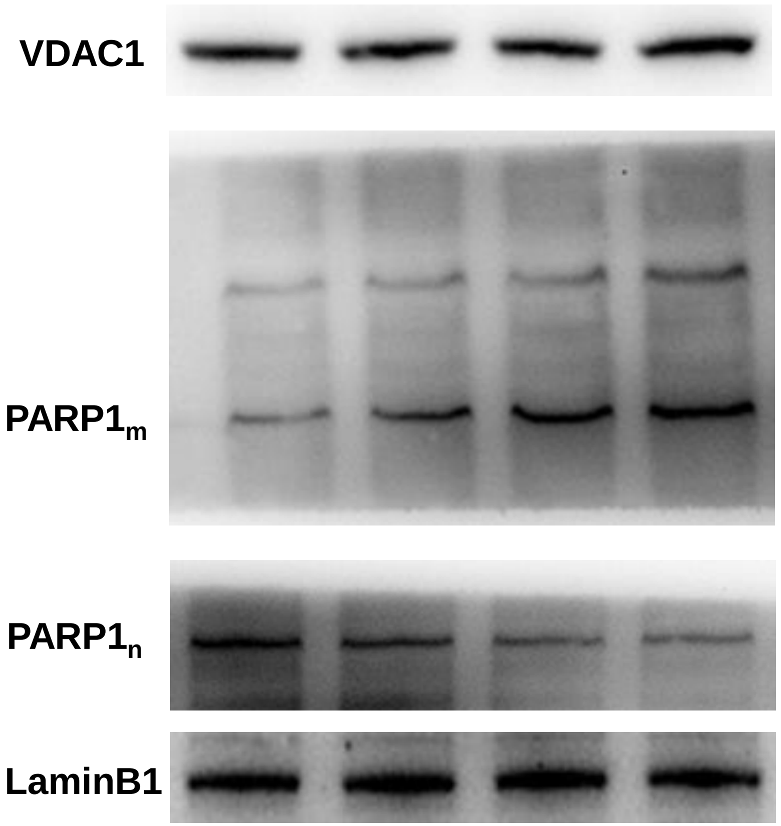

Figure 3D

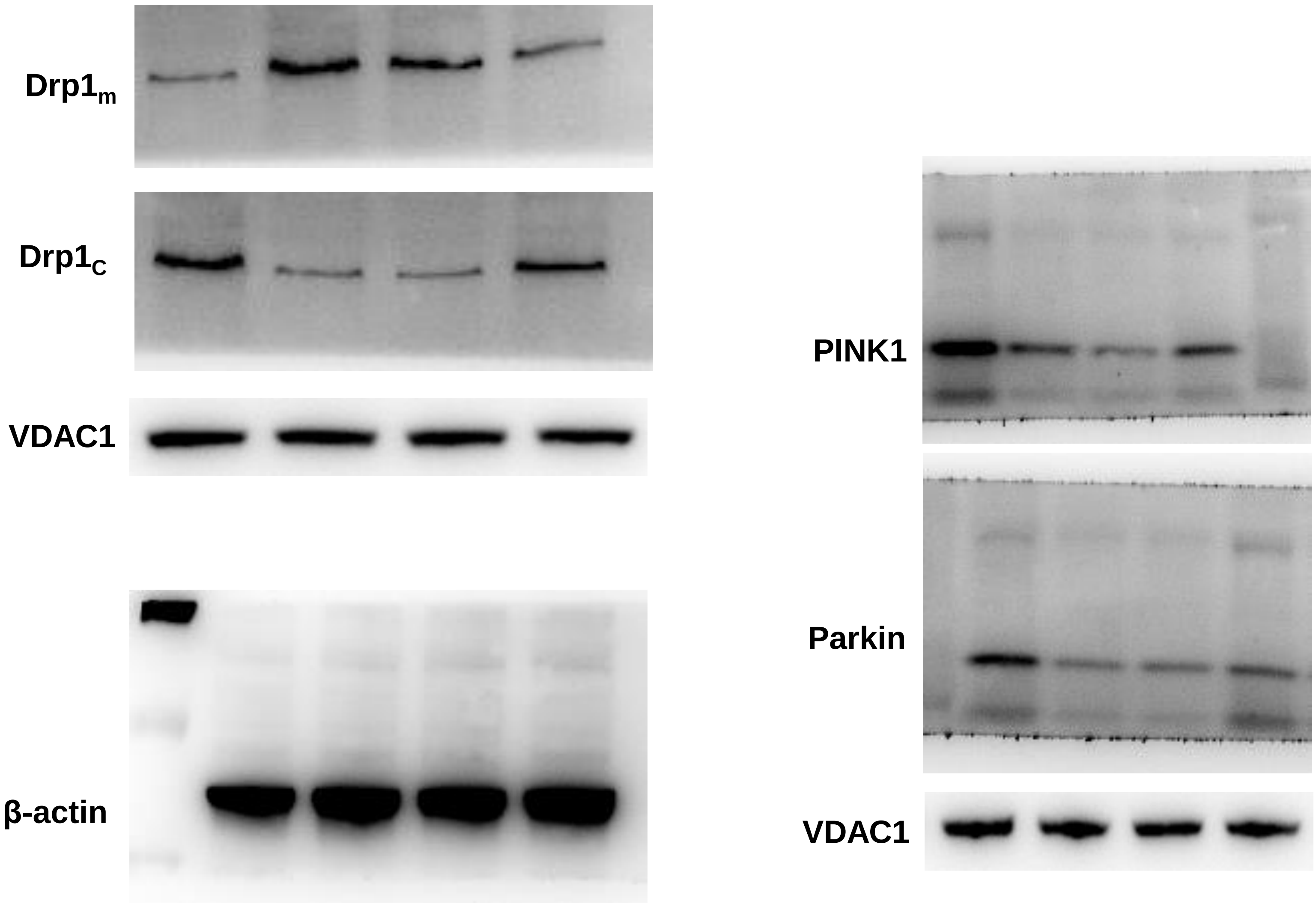

Figure 5A

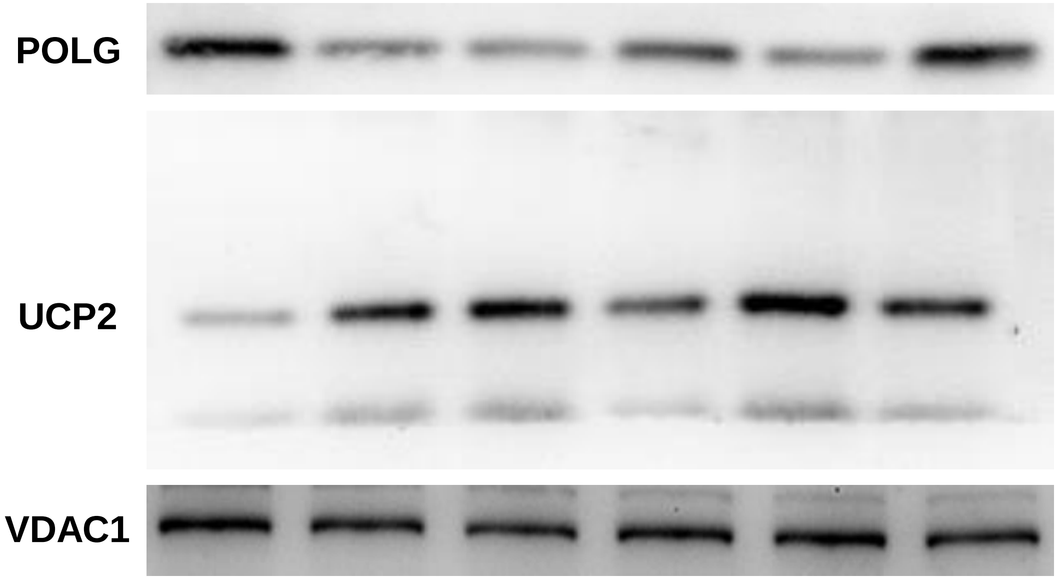

Figure 5G

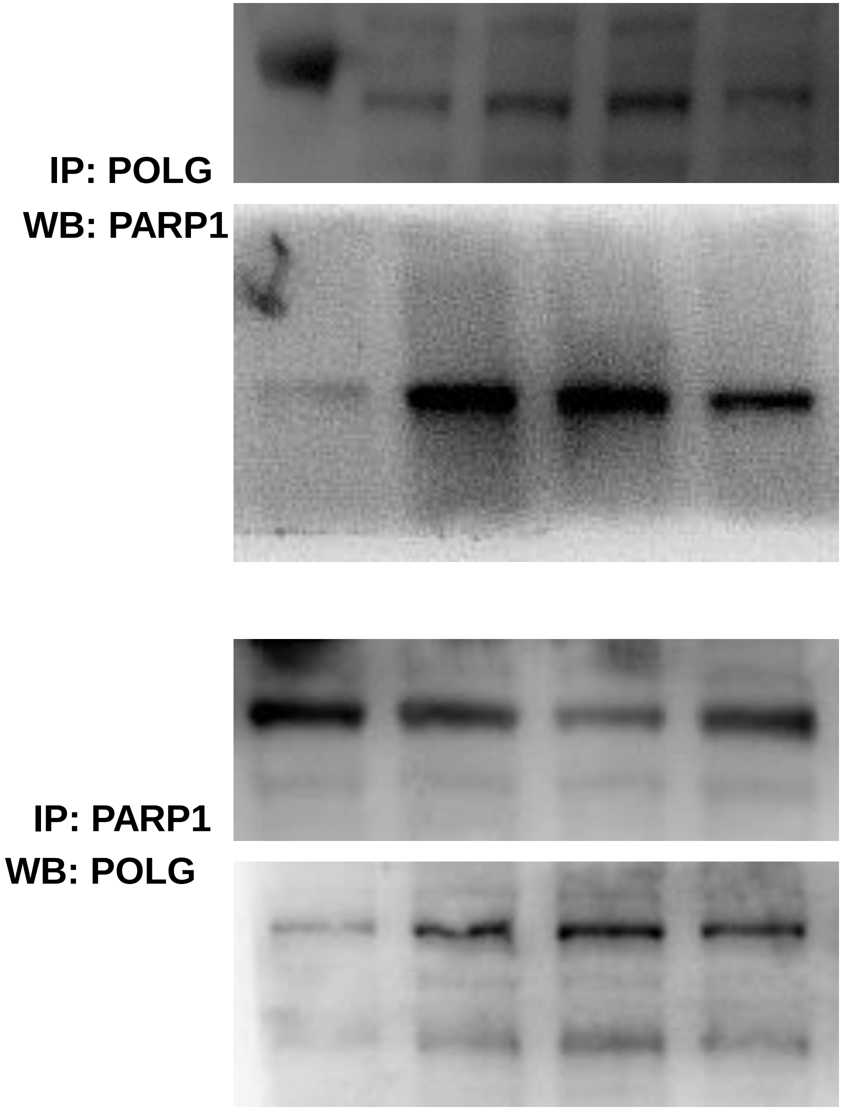

Figure 5H

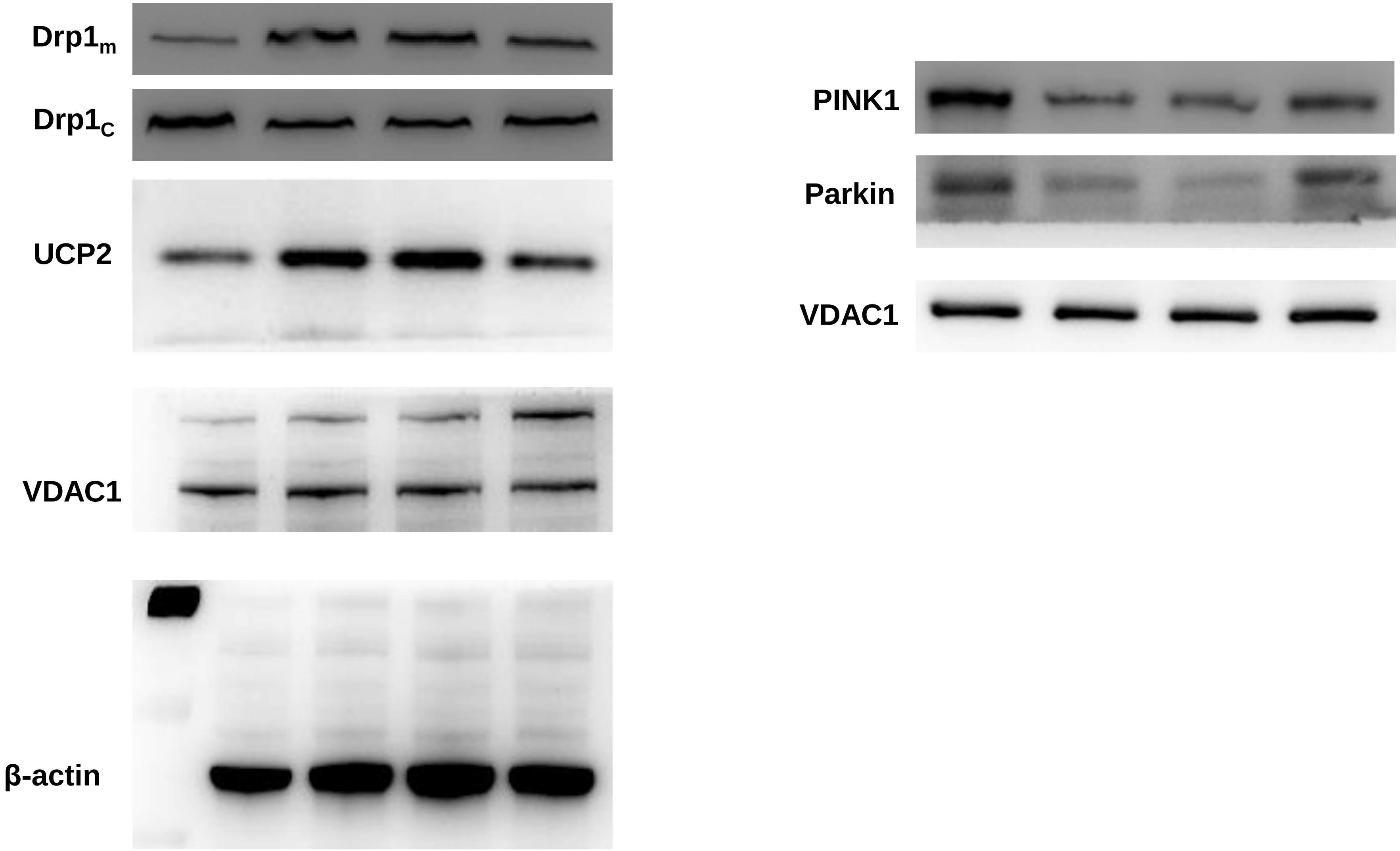

Supplement: Supplementary file 2 — Full and uncropped western blots [file 41419_2023_6113_MOESM2_ESM.pdf]
